# Identification of Arsenic Direct-Binding Proteins in Acute Promyelocytic Leukaemia Cells

**DOI:** 10.3390/ijms161125994

**Published:** 2015-11-10

**Authors:** Tao Zhang, Haojie Lu, Weijun Li, Ronggui Hu, Zi Chen

**Affiliations:** 1 Department of Laboratory Medicine, Huashan Hospital, Fudan University, 12 Central Urumqi Road, Shanghai 200040, China; shmuzt@126.com; 2 Shanghai Cancer Center and Key Laboratory of Glycoconjugates Research Ministry of Public Health, Fudan University, Shanghai 200032, China; luhaojie@fudan.edu.cn; 3 State Key Laboratory of Molecular Biology, Institute of Biochemistry and Cell Biology, University of Chinese Academy of Sciences, 320 Yueyang Road, Shanghai 200031, China; drchenzi2@163.com; 4 Cancer Research Center, SIBS-Xuhui Central Hospital, Institute of Biochemistry and Cell Biology, Shanghai Institutes for Biological Sciences, Chinese Academy of Sciences, 320 Yueyang Road, Shanghai 200031, China; 5 Department of Hematology, Huashan Hospital, Fudan University, Shanghai 200040, China

**Keywords:** arsenic-binding protein, arsenic-biotin, acute promyelocytic leukaemia, LC-MS/MS, pyruvate kinase M2

## Abstract

The identification of arsenic direct-binding proteins is essential for determining the mechanism by which arsenic trioxide achieves its chemotherapeutic effects. At least two cysteines close together in the amino acid sequence are crucial to the binding of arsenic and essential to the identification of arsenic-binding proteins. In the present study, arsenic binding proteins were pulled down with streptavidin and identified using a liquid chromatograph-mass spectrometer (LC-MS/MS). More than 40 arsenic-binding proteins were separated, and redox-related proteins, glutathione S-transferase P1 (GSTP1), heat shock 70 kDa protein 9 (HSPA9) and pyruvate kinase M2 (PKM2), were further studied using binding assays *in vitro*. Notably, PKM2 has a high affinity for arsenic. In contrast to PKM2, GSTP1and HSPA9 did not combine with arsenic directly *in vitro*. These observations suggest that arsenic-mediated acute promyelocytic leukaemia (APL) suppressive effects involve PKM2. In summary, we identified several arsenic binding proteins in APL cells and investigated the therapeutic mechanisms of arsenic trioxide for APL. Further investigation into specific signal pathways by which PKM2 mediates APL developments may lead to a better understanding of arsenic effects on APL.

## 1. Introduction

Although arsenic can be a poison and may cause serious health problems with chronic exposure, arsenic has been used as a cancer chemotherapeutic agent for many years due to its significant medicinal effects. Acute promyelocytic leukaemia (APL) is an acute myeloid leukaemia associated with a recurrent abnormal chromosomal translocation of t(15;17) and the subsequent expression of a novel fusion protein, PML-RARα [1]. In many clinical trials, both newly diagnosed and all-trans retinoic acid resistant APL patients can achieve complete remission after arsenic trioxide (As_2_O_3_) treatment [2,3,4]. APL is particularly sensitive to As_2_O_3_, which makes As_2_O_3_ a promising cure for APL patients, even as a single agent [5]. As_2_O_3_ specifically induces the degradation of PML-RARα, and consequently, the differentiation of leukaemic cells [6,7]. Twenty-four arsenic-binding proteins in the membrane fraction were identified in human lung cancer cells, whereas two additional arsenic-binding proteins, pyruvate kinase M2 (PKM2) and beta-tubulin, were identified in human breast cancer cells. Using direct cross-linking with cellular targets, beta-tubulin has been proven to be a target protein in acute myeloid leukaemia [8,9,10]. However, the medicinal effects of As_2_O_3_ are not explained solely by the degradation of the PML-RARα fusion protein in APL cells. Inhibition of other enzymes and proteins that directly bind to arsenic may also cause the therapeutic effect.

The identification of arsenic direct-binding proteins is essential for determining the mechanism by which As_2_O_3_ achieves its chemotherapeutic effects. Many arsenic-binding proteins have been identified in mammalian cells, and the biological functions of these arsenic-binding proteins are related to the formation of adducts between closely spaced SH groups in cysteine residues [11]. At least two cysteines close together in the amino acid sequence are crucial to the binding of arsenic and crucial to identify the arsenic-binding proteins [12]. The direct binding of arsenic through cysteines residues may suppress the functions of arsenic-binding proteins [13]. Therefore, it is important to identify the arsenic-binding proteins that target cysteine residues.

Our present study focuses on the identification of arsenic-binding proteins in NB4 human APL cells using biotin-conjugated trivalent arsenic. We identified novel candidate proteins that directly bind to As_2_O_3_.

## 2. Results

### 2.1. Arsenic-Biotin Inhibits APL Cell Proliferation

**Figure 1:**
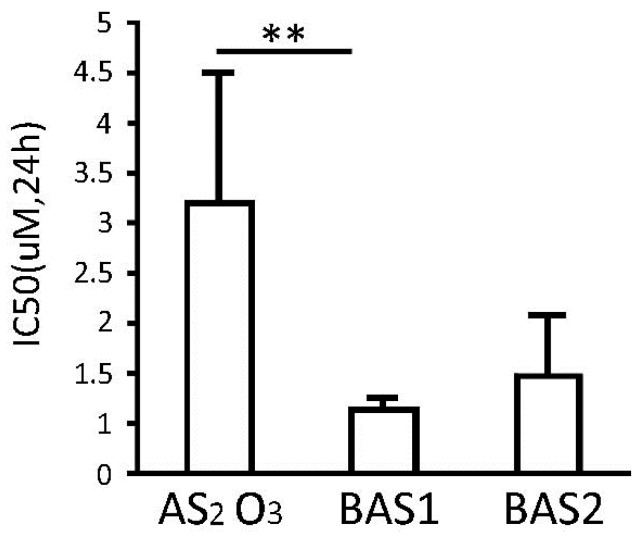
Effect of As_2_O_3_, biotin-As I (BAS1) and biotin-As II (BAS2) on NB4 cell growth. Each value represents the mean ± SD (*n* = 6) of three independent experiments. ** *p* < 0.01.

Different concentrations of As_2_O_3,_ biotin-As I and biotin-As II were utilised to assess the effects of arsenic-biotin conjugates on the growth inhibition of the NB4 cells. We established a cubic equation linking the cell inhibition rate to the drug concentration, and obtained IC_50_ values for As_2_O_3_, biotin-As I, and biotin-As II at 24 h of 2.71 ± 2.89 μM, 0.64 ± 0.10 μM and 0.98 ± 0.60 μM, respectively (Figure 1). Biotin-As I showed the greatest effect, with a significant difference from As_2_O_3_ (*p* < 0.01). As a result, 1 μM biotin-As I was used for subsequent experiments.

### 2.2. Measurement of Arsenic-Binding Proteins by Western Blot

**Figure 2:**
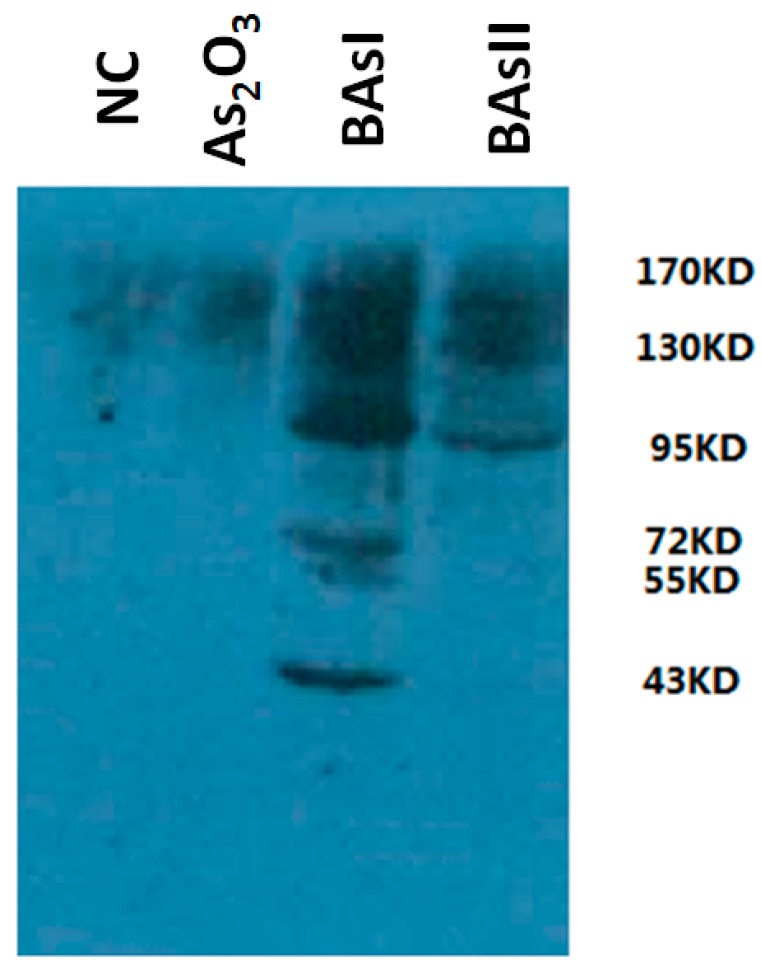
Detection of arsenic-biotin conjugating proteins in NB4 cells. NC (negative control).

To identify the arsenic-binding proteins pulled down with streptavidin, a fraction of the NB4 cell lysate was combined with arsenic-bound resin, and the bound and unbound proteins were identified by Western blot. Figure 2 shows the proteins specifically bound in the arsenic-biotin elution compared to the negative control and As_2_O_3_, demonstrating the high affinity of arsenic-binding proteins.

The arsenic binding proteins were pulled down with streptavidin. Many protein bands identified in the elution fraction of the NB4 cells treated with arsenic-biotin were compared to the NB4 cells treated with As_2_O_3_ and the negative control.

### 2.3. Identification and Characterisation of Arsenic Binding Proteins

In the present study, 2-DE was used to identify and analyse the arsenic-binding proteins based on the observation that two vicinal cysteines can bind to arsenic. Over 40 proteins contain at least two nearby cysteines compared to the negative control. These proteins are divided into 7 categories based on their functions (Table 1).

**Table 1:** Arsenic-binding proteins identified by MS.

Function	Protein
Redox-related proteins	GSTP1, PKM2, HSPA9, LEG1 Galectin-1, AT8B4, XRRA1, GAPDH, LCE1B, TET2.
DNA-dependent transcription	RL12, Med29, DNA topoisomerase 1, DNA ligase 1, RL21, RS4X, RL23.
Regulation of glycometabolism and lipid metabolism	SGSM2, RREB1, NFYC, FBN1, AL1A3, haemoglobin, ACSM4.
G-protein coupled receptor family	LPAR1, GPCRs, RXFP2.
Inflammation response	Eosinophil peroxidase, NK-tumour recognition protein, RXFP2, DHX8.
Cell proliferation and cell cycle	DNLI1, UBP2, LAMB2, Galectin-1, HNRPR, PCDGI, HTRA1, Cytochrome P450.
Proteasome homeostasis	UBXN1, PIAS3, ML12, RING finger protein 144A-B.

### 2.4. Confirmation of Binding of Redox-Related Proteins to Arsenic

**Figure 3:**
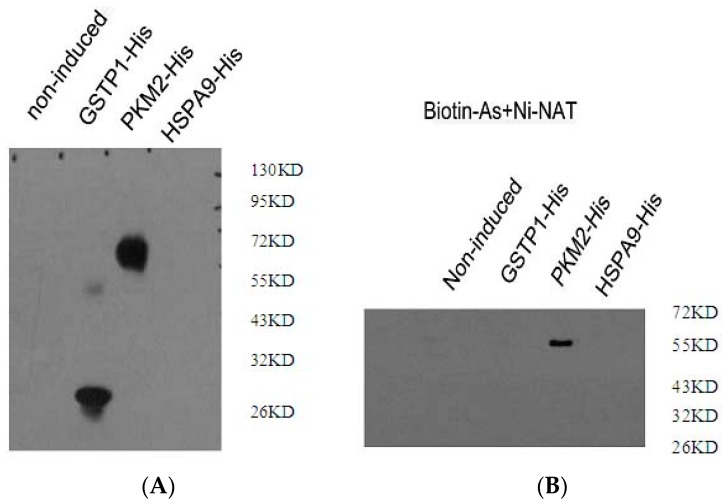
Detection of the interaction between arsenic-biotin and the protein *in vitro*. (**A**) Binding assay with His antibody; (**B**) binding assay with biotin antibody.

In this study, more than two cysteines close together were required for the identification of arsenic binding proteins. Redox-related proteins (GSTP1, PKM2 and HSPA9) were identified using this criterion. To confirm the direct combination of redox-related proteins (GSTP1, PKM2 and HSPA9) to arsenic, the recombinant plasmids pET-22b-GSTP1, pET-22b-PKM2 and pET-22b-HSPA9 were tested in a binding assay using a His and biotin antibody. Figure 3 shows that no protein specifically bound to HSPA9 with the His/biotin antibody compared to the negative control. Although GSTP1 was detected with the His antibody, there was a negative result with the biotin antibody. After pull-down with streptavidin, PKM2 was confirmed with the His and biotin antibody. These data indicate that PKM2 is an arsenic-binding protein in NB4 cells.

### 2.5. Arsenic-Biotin Suppressed PKM2 Activity

**Figure 4:**
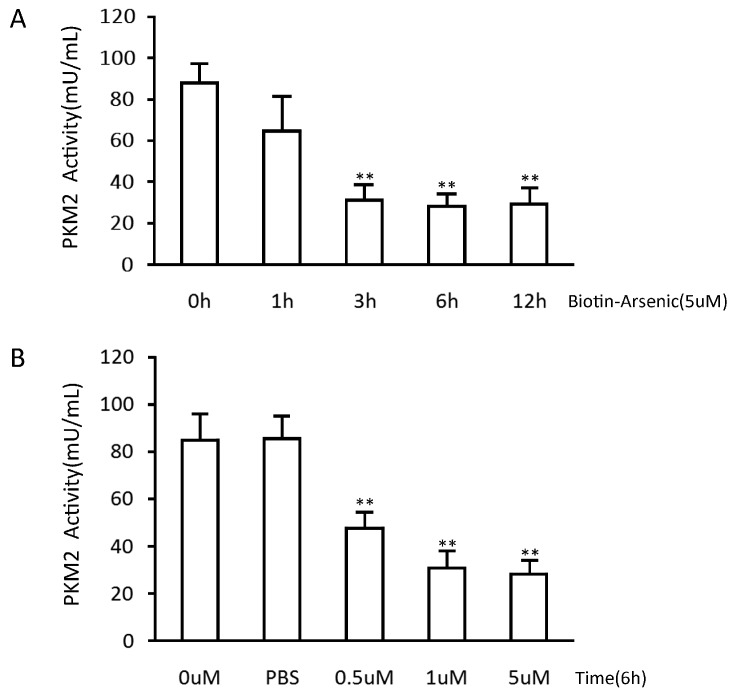
Arsenic-biotin suppresses PKM2 activity in NB4 cells. (**A**) Arsenic-biotin suppresses PKM2 activity in a time-dependent manner; (**B**) The changes in PKM2 activity in the cells treated with different concentrations of arsenic-biotin for 6 h. The PBS group was used as a negative control. The average of three measurements in separate experiments is shown with the SD; ** *p* < 0.001.

To verify the function of the arsenic-biotin on PKM2, we compared the PKM2 activities between the arsenic-biotin treated NB4 cells and the negative control. The PKM2 activity was 67% lower than the negative control after treatment with arsenic-biotin (Figure 4). Therefore, arsenic-biotin partially inhibits NB4 cell growth by reducing PKM2 activity.

## 3. Discussion

Arsenic has garnered attention as a multi-functional anticancer chemical. However, the mechanism by which arsenic exerts its clinical efficiency in APL is not fully understood. Although researchers found that arsenic specifically induces degradation of the PML-RARα fusion protein in APL, as well as its normal PML counterpart [14,15,16,17], this pathway cannot be the only molecular basis of arsenic in APL. To determine whether arsenic directly alters the biochemical features of APL cells, we examined the arsenic direct-binding proteins in NB4 cells. Notably, biotin-As I was significantly more potent than As_2_O_3_ in NB4 cells. One possibility is that biotin increases the ability of As_2_O_3_ to combine with proteins. In the anti-leukaemia mechanisms of arsenic compounds, arsenic is the main active element. However, different arsenic compounds show different activities towards leukaemia. In addition to As_2_O_3_, tetra-arsenic tetra-sulphide (As_4_S_4_), melarsoprol and phenylarsine oxide also have anti-leukaemia effects [18,19,20]. However, the mechanisms of anti-leukaemia effects are unknown. In the present study, more than 40 arsenic-binding proteins were separated, and the redox-related proteins GSTP1, HSPA9 and PKM2, were further investigated using a binding assay.

Because cysteine residues are crucial for the activity of many redox enzymes, it is possible that the interaction between arsenic and cysteine residues may suppress the function of the arsenic-binding proteins. Proteins with high affinity for arsenic that are related to redox-related proteins were the focus of this study.

The activity of GSTP1 is correlated with As_2_O_3_ sensitivity [21]. Some studies indicated that GSTP1-1 had an inhibitory impact on the As_2_O_3_ activity in lymphoma cells. Bernardini S *et al.* found that APL cells treated with As_2_O_3_ showed a high level of oxidative stress that was related to an increase of cellular GSH levels [22]. GSTP1 polymerisation was detectable and was followed by an increased apoptotic rate of the leukaemia cells. GSTP1 polymerisation was not found in As_2_O_3_-resistant cells. Notably, human GSTP1-1 has four cysteine residues, of which Cys47 displays a low p*K*_a_ value and is the most reactive [23]. Also, Cys47 is the target modified by a variety of compounds or drugs with different chemical natures. Therefore, GSTP1 was selected in this study because it is an important arsenic-binding protein in APL cells.

Leukaemia cells have increased glycolytic activity [24,25,26]. Recent studies demonstrated that PKM2 functions as a protein kinase and plays a potential role in tumour metabolism and growth [27]. The glycolytic enzyme activity of PKM2 is regulated by various oncogenes and tumour suppressors. These regulations modulate aerobic glycolysis. Favouring a shift of the dimer-tetramer dynamic towards dimerisation is critical for PKM2 to promote oncogenic anaerobic glycolysis (“Warburg effect”) leading to tumourigenesis and cancer cell proliferation [28,29]. Recently, PKM2 has been reported to play a critical role in protecting against oxidative stress in primary haematopoietic cells. PKM2 deletion enhances oxidative phosphorylation at the expense of glycolysis and biomass intermediates in primary haematopoietic progenitors [30]. Because PKM2 is important for leukaemia initiation, we tested whether PKM2 could be combined with arsenic directly because arsenic binds proteins neighbouring cysteine residues.

Heat shock 70-kDa protein 9 (HSPA9) has been localised to chromosome 5, band q31, which is a region frequently deleted in some kinds of myeloid neoplasms. HSPA9 has been proved to be a candidate tumour suppressor gene [31]. One study showed that knockdown of HSPA9 delayed the maturation of erythroid precursors in human hematopoietic cells and reduced the erythroid precursors, B lymphocytes, and megakaryocyte/erythrocyte progenitors in a murine model [32]. It is interesting to note that HSPA9 has a relationship with intracellular nitric oxide (NO), in which nitronate cysteine residues at the thiol group generate nitrosocysteine [33]. Therefore, HSPA9 was also investigated as a possible arsenic-binding protein in our study.

In this study, we investigated the characteristics of redox-related proteins (GSTP1, HSPA9 and PKM2) combined with arsenic. We performed an arsenic-biotin combination test to detect arsenic-biotin proteins *in vitro*. Notably, PKM2 has a high affinity for arsenic. In contrast to PKM2, GSTP1and HSPA9 did not combine with arsenic directly *in vitro*. These observations suggest that arsenic-mediated APL suppressive effects could be relevant to PKM2. Prior to our present study, PKM2 had not been investigated as an arsenic direct-binding protein in APL cells. As predicted, arsenic-biotin had an interesting effect on PKM2 activity. Arsenic-biotin significantly inhibited the PKM2 activity in NB4 cells.

In summary, we identified several arsenic direct-binding proteins in APL cells and investigated the therapeutic effect of arsenic for APL. Investigation of specific signalling pathways in which PKM2 mediates APL development may lead to further understanding of arsenic’s effects on APL.

## 4. Materials and Methods

### 4.1. Chemicals and Cells

The arsenic-biotin conjugates were kindly provided by Professor Ronggui Hu. As_2_O_3_ (Sigma, Chemical Co., St. Louis, MO, USA) was dissolved in 1 M NaOH as a stock solution. The human APL cell line NB4 (kindly provided by Professor Jingyi Shi of Shanghai Institute of Haematology, Rui-Jin Hospital, Shanghai, China) was maintained in RPMI-1640 containing 10% foetal bovine serum (Invitrogen Ltd, Carlsbad, CA, USA). Cells were cultured at 37 °C in a humidified atmosphere containing 5% CO_2_.

### 4.2. MTT Assay

To verify the effect of arsenic-biotin conjugates on the viability of NB4 cells, an MTT assay was performed. Briefly, NB4 cells were seeded in 96-well plates (Corning, St. Louis, MO, USA) at a density of 2 × 10^4^ cells/well and cultured overnight. Then, the media were replaced with 100 μL fresh media containing different concentration of As_2_O_3_, biotin-As I or biotin- As II (0.01, 0.05, 0.1, 0.5, and 1 µmol/L) and the cells were cultured for 24 h. At the end of the incubation, 10 μL of the MTT solution (5 mg/mL) was added to each well and the plates were incubated for 4 h at 37 °C. Then, 50 μL of DMSO (Sigma) was added to each well. The optical density for absorbance values each experiment was assessed with a Microplate Reader 550 (Bio-Rad, Hercules, CA, USA) at 570 nm.

### 4.3. Identification of Arsenic Direct-Binding Proteins

First, NB4 cells were blocked with sulphhydryls on protein, and then cells were treated with arsenic -biotin conjugates for 12 h. The NB4 cells were washed with PBS and urea buffer. Then the cell lysate was digested with trypsin at 37 °C overnight. The cell lysate was mixed with streptavidin resin (100 μL) after inactivation with 1% formic acid. The resin was agitated at 4 °C for 3 h, and then was washed and resuspended in loading buffer before the separation on gel. All proteins pulled down with streptavidin were collected. The arsenic direct-binding proteins were determined by comparing the elution fraction of the negative control with the NB4 cells treated with arsenic-biotin. These protein bands were cut into slices for LC MS/MS analysis.

### 4.4. Proteins Extraction and LC-MS/MS

NB4 cell lysate treated with arsenic-biotin was blocked with *N*-ethylmaleimide, and then the proteins were enriched using streptavidin beads. The peptides were concentrated with streptavidin after trypsin digestion and then analysed by LC-MS/MS.

The nano-LC MS/MS experiments were performed on an LTQ-Orbitrap mass spectrometer (ThermoFisher, San Jose, CA, USA) coupled with an LC-20AD nano-flow HPLC system (Shimadzu, Tokyo, Japan). The sample was separated with a PICOFRIT C18 reverse-phase column (New Objective Inc., Woburn, MA, USA) and the flow rate was 300 nL/min. The mobile phases included phase A (2% acetonitrile with 0.1% formic acid) and phase B (95% acetonitrile with 0.1% formic acid). A 90-min linear gradient from 5% to 45% phase B was used to acquire separation. The mass spectrometer was done in a data-dependent mode. Each cycle of duty consisted of one full MS survey scan in the mass range 350~1800 Da with a high resolution power Orbitrap section, followed by MS2 for the 10 strongest peaks using the LTQ section. Peptides were fragmented in the LTQ section using collision-induced dissociation (CID) with helium, and the normalised collision energy value was set at 35%.

### 4.5. Database Search

Proteins were searched using the BioWorks 3.3.1 sp1 software (ThermoFisher, San Jose, CA, USA) against the Swissprot databases for humans using the TurboSequest search engine v.27 with the following criteria: 2 possible missed cleavage sites, peptide mass tolerance of 20 ppm, fragment mass tolerance of 1.00 Da, and 15 Da shift for oxidised Met were regarded as possible modifications. The same filtration criteria was used to determine the acceptance criteria for peptide identification by searching the files against a reversed Swissprot databases. Using these stringent filtration criteria, the rate of false positive identification was less than 5%, thereby increasing the confidence of the identified proteins.

### 4.6. Arsenic-Binding Protein Analyses

GSTP1, PKM2 and HSPA9 genes were amplified by PCR with specific primers based on the encoding gene sequence. The amplified genes were recovered and cloned into the pET-22b expression plasmid. There recombinant plasmids were named pET-22b-GSTP1, pET-22b-PKM2 and pET-22b-HSPA9. All constructed vectors were confirmed by DNA sequencing. The binding assay was performed as follows: first, the protein was deoxidised at 4 °C for 20 min, and then the protein was desalted and eluted at 200 μL/tube for SDS-PAGE. A total of 5 μg protein was incubated with 10 μM arsenic-biotin for 1 h, and then the mixture was rotated at 4 °C for 2 h. The resin was resuspended in 50 μL loading buffer, and then 15 μL Ni-NTA beads were added. Then the mixture was rotated at 4 °C for 2 h and the supernatant was transferred to nitrocellulose membranes after SDS–PAGE. The membranes were incubated with His and biotin primary antibody overnight at 4 °C, and then the membranes were washed and incubated with the secondary antibody.

### 4.7. Pyruvate Kinase Activity Assay

To observe the effect of arsenic-biotin on the intracellular PKM2 activity, we used arsenic-biotin to treat NB4 cells with a concentration gradient (0, 0.5, 1 or 5 µM for 6 h) or a time gradient (5 µM for 0.5, 1, 3, 6 or 12 h). NB4 cells in logarithmic phase were cultured in 6-well plates at a density of 1 × 10^5^ cells/well in 2 mL RPMI-1640 + 10% fetal calf serum (FBS). Arsenic-biotin was added to the final concentration and the cells were cultured at 37 °C and 5% CO_2_. The cells were collected by centrifugation at 3000× *g* and subjected to PKM2 activity assay using the Pyruvate Kinase Activity Colorimetric Assay Kit (BioVision, Milpitas, CA, USA) according to the manufacturer’s instructions, followed by data analysis.
